# Prevalence of Hearing Loss among School-Age Children in the North of Iran

**DOI:** 10.22038/ijorl.2019.36090.2191

**Published:** 2020-03

**Authors:** Mir Mohammad Jalali, Fatemeh Nezamdoust, Hedieh Ramezani, Masomeh Pastadast

**Affiliations:** 1 *Otorhinolaryngology Research Center, Department of Otolaryngology and Head and Neck Surgery, School of Medicine, Guilan University of Medical Sciences, Rasht, Iran. *

**Keywords:** Children, Hearing evaluation, Pediatrics, Screening

## Abstract

**Introduction:**

The present study aimed to investigate the audiological profiles of elementary school-age children in Rasht, Iran, and estimate the prevalence of hearing impairments in this population**.**

**Materials and Methods:**

In this cross-sectional descriptive-analytical study, the hearing threshold was screened using pure tone audiometry (PTA). Hearing impairment was defined as equal to or higher than 20 dB HL. Results of the hearing thresholds were separately reported in the left or right ears and better or worse ears. Logistic regression tests were used to investigate the association between hearing loss and possible risk factors. In this study, all the analyses were conducted using SPSS software (version 21).

**Results:**

The present study was carried out on a total of 2019 children. Mean age of the participants was reported as 9.66±1.66 years. Based on low-frequency pure-tone average, the prevalence rates of hearing loss > 15 dB in the right and left ears were reported as 1.94% and 1.68%, respectively. The high-frequency hearing loss > 15 dB in the right and left ears was obtained at 1.14% and 1.04%, respectively. Prevalence rate of hearing loss (in all frequencies) in boys was higher than that in girls. There was a strong association between a history of otitis media and sensorineural or conductive hearing loss (adjusted odds ratio reported as 12.2 and 8.1, respectively).

**Conclusion:**

In this study, the rate of hearing loss in the participants was approximately 2%. It was concluded that the screening of hearing loss in children is necessary for the identification and management of these children as early as possible. It is recommended to perform further trials to investigate the impact of different causes on childhood hearing impairment.

## Introduction

Hearing impairment has adverse effects on a child academic performance, as well as development of appropriate speech, language, and psychosocial abilities (1). Any type of hearing loss can delay the children progress at school, resulting in learning and behavioral problems. Although hearing loss reduces learning skills in the first year of primary school, this effect will be considerable in the third or fourth grade. This difficulty may be as a result of complex relationship among language, social interaction, verbal communications and auditory information. According to the literature, it was demonstrated that children with a hearing loss have limited educational and vocational attainment. Risks of physical, social, emotional, and sexual abuse and even fatal injuries are higher among these children (2).

The hearing screening programs have been used as essential tools to find hearing-impaired children in developed countries. Routine screening of hearing impairment in school-age children should be considered in low- and middle-income countries, as it can be more readily implemented than universal neonatal screening (2). In children under 15 years of age, 60% of hearing loss is attributed to preventable causes. Early diagnosis and intervention are essential to minimize the impact of hearing loss on child development and academic performance (3). 

The World Health Organization (WHO) estimates the prevalence of mild hearing impairment in the region of the Middle East and North Africa region at 4.5% (Prevalence range: 2.0-10.4%) and 2.8% (Prevalence range: 1.2-6.7%) for boys and girls within the age range of 5-14 years, respectively. Corresponding prevalence rates ≥ moderate hearing impairment are reported as 0.8% and 0.5% for boys and girls, respectively (4). 

The Iranian national program for ear health and hearing is currently undergoing countrywide expansion (in the Ministry of Health and Medical Education). Primary objective of this program is to screen hearing impairment in 100% of neonates in the country. Another focus of the program is to increase the coverage of hearing screening for children within the age range of 3-6 years by 30% in the country (5). Data on the magnitude of hearing impairment are limited and not up-to-date in Iran. Several previous estimations of the prevalence rate from Iran using the WHO definition of hearing impairment were within the rage of 0.3-9.8%; however, the most recent estimation was obtained over 10 years ago(6-10). 

Sarafraz et al. (6) evaluated the audiologic reports of 785 school-children retrospectively in Ilam province, Iran. In the aforementioned study, 77 (9.8%) subjects in the first and second grades had hearing loss. Among hearing-impaired children, 44 (57.1%) and 33 (42.9%) cases were boys and girls, respectively (P<0.05). In a study carried out by Absalan et al. (7), 1500 students were screened for hearing impairment from elementary schools in Zahedan, Iran. The results showed the prevalence rate of hearing impairment as 8.2% in the students. There was no significant difference between the prevalence of hearing impairment in boys and girls. 

Hajilo et al. (8) assessed hearing impairment in Ardabil, Iran, using questionnaire clinical audiometry. It was observed that out of 2497 (0.3%) children, 11 subjects (age<14 years) suffered from hearing impairment. There was no significant difference between the two genders in this regard (P>0.05). Difference of reported prevalence rates could be due to sampling errors or differences in age range, analysis methods, and screening methodologies (9). Although several studies showed hearing loss among school children as a considerable health problem, there is no accurate estimation of hearing loss prevalence among Iranian children. For strengthening present national efforts and adapting to the WHO global disability action plan: Better Health for Persons with Disabilities 2014–2021, it is needed to monitor the progress of this plan and identify areas for further policy improvements. The present study aimed to investigate the audiological profiles of elementary school-age children in Rasht, Iran, and estimate the prevalence of hearing impairments in this population.

## Materials and Methods

This cross-sectional study was designed to evaluate the prevalence of hearing loss among the boys and girls of elementary school-age in urban areas in Rasht, northern Iran. Rasht is the capital of Guilan province in Iran with a population of 920,000. The study protocol was approved by the Review Boards of Guilan University of Medical Sciences, Guilan, Iran, (approval id: IR.GUMS.REC.1394.135) and complied with the principles outlined in the Helsinki Declaration. 

Sampling was performed using multistage random cluster sampling method. Firstly, 15 schools were randomly selected from a list of elementary schools in the urban areas of Rasht. Then, sampling occurred in classes randomly chosen from each grade in the selected schools. A total of 2100 students were selected from all 6- to 13-year-old children during January and April 2015. Informed consent was obtained from the parents of the participants. Overall, 81 parents would not like to participate in this study; therefore, 2019 children (i.e., 1046 boys and 973 girls) were included in data analysis. Number of boys and girls participated in this study was proportional to the number of schools for boys and girls.

Screening for hearing impairment was conducted by an expert audiologist. A structured questionnaire was distributed to the parents for the determination of the medical and family history of the children and evaluation of their socioeconomic levels. A general medical examination was carried out by two trained interviewers. The ear canals were examined with an otoscope. Foreign bodies, debris, and impacted cerumen were removed. Any children who were diagnosed with acute chronic otitis media or otitis media with effusion were not excluded from the study. The quietest areas in each school with the noise level lower than 45 dB were chosen to perform audiometric tests. A duly calibrated pure-tone audiometer was used with TDH-39 earphones and Audiocups for extra attenuation. The children who failed were referred to Amiralmomenin hospital for otorhinolaryngologic assessment and comprehensive audiometric evaluation. The failure was considered a pure-tone average > 15 dB HL at frequencies 0.5-4.0 kHz. The hearing threshold level was calculated by the average of the frequencies of 500, 1000, 2000, and 4000 Hz. Pure-tone average (PTA) was classified into low-frequency pure-tone average (LPTA) and high-frequency pure-tone average (HPTA), which was the mean hearing threshold within the frequency ranges of 0.5-2 and 4-8 kHz, respectively. 

As shown in Table 1, the LPTA and HPTA were classified into three groups (5), including unilateral (<20 dB HL in the better ear and ≥35 dB HL in the worse ear), mild (20-34 dB HL), and ≥ moderate (≥35 dB HL). Hearing loss was categorized as sensorineural and conductive if the air-bone gaps were < 15 and > 15 dB, respectively. Mixed hearing loss was considered if both air-bone gap and bone conduction thresholds were > 15 dB. The hearing level was reported in the left and right, as well as better and worse ears.

**Table 1:** Hearing impairment categories

**Hearing impairment category**	**Better ear hearing level (dB HL)**
**Unilateral**	<20 in the better ear ≥35 in the worse ear
**Mild**	20-34
**Moderate**	≥35

Statistical analysis

Using the expected prevalence of hearing impairment of 9.8% (4), error margin of 16%, and design effect factor of 1.5, as well as assuming random sampling with a 95% confidence interval (CI), it was estimated that a sample size of 2094 children would be enough to determine the prevalence of hearing impairment among the children of elementary schools in Iran. Prevalence of hearing loss was reported in percentages and their 95% CIs. Logistic regression tests were used to examine the relationship between hearing loss and possible risk factors. 

The results were also reported from subgroup analyses. The data were analyzed using SPSS software (version 21)..

## Results

A total of 2019 school-age children participated in the present study. Mean age of the participants was reported as 9.66±1.66 years (age range: 6-13 years). In addition, 1046 (51.8%) and 973 (48.2%) subjects were boys and girls, respectively. Most of the children were between 8 and 12 years (86.8%). Both ears of all the cases were screened; therefore, 4038 ears were assessed in this study. Table 2 tabulates the demographic characteristics of the participants. 

Among the children, 42 subjects failed in one or both ears and were referred for comprehensive diagnostic audiometry. Moreover, 27 (i.e., 15 males and 12 females) cases were diagnosed with a hearing loss, which indicated the prevalence rate of hearing loss as 1.2%. The obtained results revealed a lower prevalence of hearing loss in the youngest age group, compared to that of the older groups (0.7% versus 1.8% and 1.1%, respectively). There was a positive history of neonatal icterus in 82 children (4.5%). In addition, 20 (1%) subjects were reported with a family history of hearing loss (Table.2). None of the participants were exposed to ototoxic drugs or environmental noise. 

**Table 2:** Demographic characteristics of study population

Variables		Total (n=2019)	Boys (n=1046)	Girls (n=973)
**Age**				
** <8 years** ** 8-10 years** ** >10 years**		144 1227 668	48 648 350	96 579 298
**Mean pure tone audiometry**		5.0±3.1	5.0±3.0	5.1±3.2
**History of neonatal icterus**		82	4	78
**History of otitis media**		1	1	0
**Positive family history**		130	47	83
**Socioeconomic status** ** Low to moderate** ** High**		1441 578	774 272	667 306

 The most common type of hearing loss, unilateral or bilateral, was conductive hearing loss (25.9% and 44.4%, respectively). The most common possible cause identified for ear examination was otitis media with effusion (57.9%) followed by chronic suppurative otitis media (15.8%). Furthermore, 8 children had sensorineural hearing loss (Table.3).

**Table 3:** Nature of hearing loss in study population

Types	Total (n=2019)	Boys (n=1046)	Girls (n=973)
**Bilateral conductive** **Bilateral sensorineural** **Unilateral conductive** **Unilateral sensorineural** **Unilateral conductive/sensorineural**	12 4 6 4 1	5 3 4 3 0	7 1 2 1 1

A history of infection (following acute otitis media and meningitis) was observed in 2 subjects. Cause of sensorineural hearing loss could not be discovered for other participants. None of the participants were reported with the mixed type. 

In addition, one child had conductive hearing loss in one ear and sensorineural hearing loss in the other ear. Binary logistic regression revealed a significant effect for a history of otitis media (OR=8.1, P=0.007). In other words, children with a positive history of 8.1 times (95% CI 1.8-36.7) were more likely to have a hearing loss than those with a negative history. However, there was no significant effect for age, gender, family history, or socioeconomic status. When the effects of various factors on conductive and sensorineural hearing loss were separately assessed, the results showed only a strong association between the history of otitis media and sensorineural hearing loss (adjusted odds ratio: 12.2; 95% CI 1.5-101.8). 

The highest prevalence of hearing loss in the right and left ear was reported at 500 Hz (i.e., 2.0% and 1.9%, respectively). The lowest prevalence of hearing loss in the right and left ear was obtained at 4000 Hz (i.e., 1.0% and 0.7%, respectively). Based on the low frequency of PTA, the hearing level of the right ear in 1.3% of children and hearing level of the left ear in 1.0% of children were ≥ 20 dB. In high-frequency PTA, different degree of hearing loss was observed in 0.7% and 0.6% of the right and left ears, respectively (Table 4). 

**Table 4:** Hearing levels based on LPTA and HPTA among student for right and left ears

**Pure tone audiometry**		**Normal**		**Mild**		**≥Moderate**	
		Percentage	95% CI	Percentage	95% CI	Percentage	95% CI
**Low frequency**	Right ear	98.6	(98.0-99.1)	1.1	(0.7-1.6)	0.2	(0.1-0.5)
	Left ear	98.9	(98.3-99.3)	0.9	(0.6-1.5)	0.1	(0.0-0.4)
**High frequency**	Right ear	99.2	(98.7-99.5)	0.4	(0.2-0.8)	0.3	(0.1-0.7)
	Left ear	99.3	(98.8-99.6)	0.3	(0.1-0.7)	0.3	(0.1-0.6)

* CI: Confidence interval

Means of LPTA and HPTA threshold in the better ear among those tested were 4.7 (range: 0-50) and 4.8 (range: 0-82.5) dB, respectively. Prevalence rates of low- and high-frequency hearing loss in the better ear were 0.8% and 0.4%, respectively. Prevalence rates of low- and high-frequency hearing loss in the worse ear were 1.5% and 1.0%, respectively. Odds ratio of hearing loss at LPTA in students with a history of otitis media to without history was 10.1 with 95% confidence intervals 1.5-56.4 and at the high frequency (HPTA) 15.5 with a 95% confidence interval 2.6-98.7.

## Discussion

Hearing impairments among school-age children in developing countries have been widely regarded as a major health problem (9, 10). Moreover, the hearing screening of school-age children is not a part of routine clinical examination in the developing countries. In the present study, hearing thresholds were assessed within a range of 500-8000 Hz among 2019 students from the elementary schools of Rasht. Overall prevalence rates of hearing loss in low and high frequencies in the worse ear were 1.5% and 1.0%, respectively. The best and poorest hearing thresholds were at 500 and 4000 Hz, respectively. Holmes et al. (11) identified similar trends and noticed that a decrease in the hearing threshold could be suggestive of noise exposure. They also observed conductive and sensorineural hearing loss in 0.9% and 0.4% of students, respectively. 

The WHO has defined a disabling hearing impairment in children younger than 15 years as a permanent unaided hearing threshold in the better ear > 30 dB HL (12). According to the WHO criteria, disabling hearing impairment was observed in about 0.1% of children. Unilateral hearing losses (37.0%, 10/27) were less common than bilateral losses. Although bilateral hearing loss causes communication difficulties in children due to adverse listening conditions, such as noisy classrooms (6), children with unilateral hearing losses have also an increased rate of grade failure and need additional academic support. Because the decibel scale is exponential, even a slight dB change in a child hearing threshold at any frequencies can have adverse effects on the hearing ability. Therefore, unilateral or bilateral hearing loss could affect academic performance and vocational outcomes (13).

Findings of the present study regarding the prevalence rate of hearing loss are consistent with the rates obtained in a study by Hajilo (8). The aforementioned population-based cross-sectional survey was conducted in the rural and urban population of Ardabil province (in the northwest of Iran). Rate of hearing impairment among children < 15 years was reported as 0.3%. However, several studies (6,7) in different provinces of Iran reported higher prevalence rates of hearing loss in school-age children. Despite the differences between the results of the present study and three previous studies, the findings of the present study are in line with global WHO estimations obtained from the Middle East and North Africa region of 2.8-4.5% (4). 

Lower prevalence of hearing impairment in the present study may be due to the fact that cerumen in the children with cerumen was removed, re-screened, and counted as normal hearing. However, cerumen was regarded as an etiology for conductive hearing loss in previous studies. Another potential reason for this finding may be an increased tendency to preschool assessment throughout the whole country in recent years. In addition, protocols used in these studies differed from the screening protocol of the present study, in which the hearing threshold at frequencies of 500 to 8000 Hz was assessed. On the other hand, the Iran Health Insurance Organization has recently increased universal health coverage to ensure that all people have access to effective health services (14). Therefore, economic issues were not barriers with respect to the timely treatment of preventable causes of hearing loss. 

A multicenter cross-sectional study was conducted by Intakorn et al. (15) to assess the bacterial etiology of acute otitis media in Thailand. In the aforementioned study, it was observed that 18% of the samples were positive for *Haemophilus influenza, and 62% of the isolates were type b. *The WHO revealed the moderate to high burden of *Haemophilus influenza* type b (Hib) in the Eastern Mediterranean countries (16). Although vaccination against Hib has not yet been a routine national immunization program in Iran, parents show greater tendency to vaccinate their children against Hib in recent years. Then, the decline of Hib incidence and its burden disease could be possible.Estimated prevalence of disabling hearing impairment in the present study may be an underestimation of the true prevalence of hearing impairment. Guilan province currently has three schools for deaf students. Therefore, it is estimated that some children with disabling hearing impairment receive educational supports in these schools. It was observed that otitis media was the most common cause of hearing impairment among school-age children. This finding was in line with the results of other studies in developing countries (6,17-20), which consider otitis media a major chronic disease in low- and middle-income countries. Implementation of pediatric hearing screening programs through Public Health Centers, as well as training of health staffs, increase the chance of identifying preventable and treatable causes of hearing impairment in children. Also, the universal childhood immunization against Hib could reduce the prevalence of childhood hearing loss with otitis media. 

According to the literature, contradictory results are shown regarding the association between hearing loss and gender. Although the result of the present study is consistent with those of previous studies (6,21) showing that male students have a nonsignificant higher prevalence of hearing loss than female students. Setoude et al. detected a more common significant hearing loss among boys, compared to that among women (32). This finding may be attributed to the pattern exposure to infections and noise-induced hearing loss in boys (22,23), which requires further investigation. 

There was no association between hearing loss and socioeconomic status. The relationship between socioeconomic status and otitis media with hearing loss is controversial, although Saim et al. (24) proposed that there was a poor hygienic condition, low immunization rate, and unnecessary use of ototoxic medications in population with low-socioeconomic status. However, some researchers (6, 25) suggest that higher socioeconomic status is associated with a higher risk of otitis media in children. It is possible that the early enrollment of children in day-care centers by these working parents intensifies the risk of cross-infection.

## Strengths and Limitations

The present study had several strengths. Firstly, the survey was population-based. Secondly, the clinical measurement of hearing impairment was conducted by an expert audiologist. Thirdly, two trained interviewers gathered the data of this study. Reported measures were also included in the survey to allow the researchers to be assured from the results. The present study had also several limitations that need to be considered. Lack of a soundproof room and possible high level of ambient noise could be the challenges of the present survey. Possible causes of hearing impairment presented in this study should be interpreted with caution. 

Causes of hearing impairment in the children could not be discovered due to the absence of comprehensive history taking, physical examination, and review of medical records. A prospective study is expected to show the true nature of the causes of hearing impairments among school-age children. All the participants were from school-age children in Rasht; accordingly, the results of this study may not be representative of all cases with the national statistics. Therefore, it is required to perform a study with a large sample size to establish a true picture of the situation in Iran. 

## Conclusion

Hearing loss is becoming a public health problem. In this study, the overall prevalence of hearing loss was reported as 1.2% in school-age children. It is recommended to perform the hearing screening of school-age children as a part of medical examinations. This program helps to identify hearing-impaired children in order to refer them for otolaryngological assessment and comprehensive audiological evaluation. It is suggested to carry out another study with a larger sample size to understand the prevalence and causes of hearing impairment among children.
